# Treatment of Established Chemotherapy-Induced Neuropathy with N-Palmitoylethanolamide: A Randomized, Double-Blind Phase II Pilot Study

**DOI:** 10.3390/cancers16244244

**Published:** 2024-12-20

**Authors:** Mellar P. Davis, Angela Ulrich, Rebecca Segal, Vinay Gudena, Kathryn J. Ruddy, Stacy D’Andre, Karthik V. Giridhar, Vamsi K. Vasireddy, Rajiv Agarwal, Abdel-Ghani Azzouqa, Paul Novotny, Shaylene McCue, Brent Bauer, Charles L. Loprinzi

**Affiliations:** 1 Levine Cancer Center, Charlotte, NC 28204, USA; mdavis@geisinger.edu; 2 Biostatistics, Mayo Clinic, Rochester, MN 55905, USAnovotny.paul@mayo.edu (P.N.);; 3 Cone Health Cancer Center, Greensboro, NC 27403, USA; liza.segal@conehealth.com (R.S.); vinay.gudena@conehealth.com (V.G.); 4 Medical Oncology, Mayo Clinic, Rochester, MN 55905, USAgiridhar.karthik@mayo.edu (K.V.G.); 5 Carle Clinic, Urbana, IL 61801, USA; 6 Department of Medicine, Vanderbilt University Medical Center, Nashville, TN 37232, USA; rajiv.agarwal@vumc.org; 7 Monument Health, Rapid City, SD 57701, USA; 8 Internal Medicine, Mayo Clinic, Rochester, MN 55905, USA

**Keywords:** chemotherapy-induced neuropathy, palmitoylethanolamide (PEA)

## Abstract

**Background:** Chemotherapy-induced peripheral neuropathy (CIPN) from oxaliplatin and taxane drugs is a bothersome toxicity. Palmitoylethanolamide (PEA) has been reported to improve myelinated nerve fiber function in patients experiencing painful CIPN. We conducted a double-blind, placebo-controlled, randomized trial of PEA in patients with established CIPN. **Methods:** Eligible patients were adults who had pain, numbness, tingling, or other symptoms of CIPN for at least three months following completion of paclitaxel, oxaliplatin, or cisplatin-based chemotherapy. Study patients were randomized to one of the two treatment groups (PEA versus placebo, both administered either once or twice daily). The CIPN20 questionnaire was assessed weekly. **Results:** A total of 17 males and 71 females participated in the study; most had neuropathy from paclitaxel. Most (85%) finished 8 weeks of treatment. There was no suggestion that either of the PEA arms did any better than the combined placebo arms. There was no signal of significant toxicity differences between the three study arms. Quality of life outcome measures were similar between the study arms, as were cognitive function evaluations. **Discussion:** PEA failed to improve established CIPN. Future trials might explore whether PEA may be effective in preventing CIPN or cognitive changes based on data that suggest it may be helpful in this situation. **Conclusions:** PEA failed to improve established chemotherapy-induced neuropathy.

## Simple Summary

Chemotherapy-induced neuropathy is a major clinical problem, with limited treatments available to try to reverse it. Some preliminary data suggested that palmitoylethanolamide (PEA) may effectively treat this condition. Pursuant to this, a placebo-controlled, double-blind, randomized phase 2 clinical trial was developed to look for evidence to support a future, more definitive phase 3 clinical trial. Eighty-nine patients participated in this clinical trial. Unfortunately, the data from the present phase 2 trial did not support the idea that PEA was useful in this situation.

## 1. Introduction

Oxaliplatin, paclitaxel, and cisplatin are important chemotherapy agents. Paclitaxel is essential in the management of lung cancer, breast cancer, and gynecological malignancies; oxaliplatin has been critical in treating gastrointestinal cancers; and cisplatin has been used in a variety of cancers [1,2,3,4,5,6,7]. Unfortunately, a significant side effect that can limit treatment with these drugs is chemotherapy-induced peripheral neuropathy (CIPN) [8,9]. Signs and symptoms of CIPN include hyperalgesia, allodynia, sporadic burning or shooting pain, muscle spasms, prickling sensations, and paradoxical numbness, largely in a stocking and glove distribution. The motor function can also be adversely affected. There can also be a loss of vibratory sense and deep tendon reflexes [10,11,12,13,14]. The prevalence of oxaliplatin neuropathy by the Surveillance Epidemiology and End Results database in patients treated for colorectal cancer at 2 years is 13.1 to 27% [15]. Increased numbers of cycles of oxaliplatin-based chemotherapy and a dose accumulation of greater than 880 milligrams/m^2^ are associated with substantial neuropathy [15]. Paclitaxel neuropathy occurs in 80% of treated patients, of which 22.4% will have persistent chronic neuropathy associated with pain [16]. The risk of neuropathy is associated with cumulative doses [17].

Palmitoylethanolamide (PEA) belongs to the endogenous anti-inflammatory lipid family of N-acylethanolamines. It is classified as an autocoid local injury antagonist amide that blocks mast-cell activation and acts as a peroxisome proliferator-activated receptor-alpha (PPAR-alpha) agonist and a transient potential receptor vanilloid type 1 (TRPV-1) modulator [18,19,20,21]. PEA has an entourage effect with the endocannabinoids, anandamide, and 2-arachidonoylglycerol by competitively blocking catabolism through fatty acid amide hydrolase [22,23,24]. PEA does not directly interact with classic cannabinoid receptors (CB1 and CB2) and thus does not produce cannabimimetic side effects; it does increase endogenous cannabinoid tone, which may contribute to analgesia and reduced neuroinflammation [23,25,26,27]. By activating PPAR-alpha, PEA blocks the upregulation of nuclear factor-kappa B and the release of inflammatory cytokines [27]. PEA re-establishes glutaminergic synaptic function, reducing neuropathic pain and improving pain-related depression in animal models [28]. Finally, PEA activates two orphan receptors, GPR 55 and GPR 119, which modulate neuroinflammatory responses to injury [29,30].

Clinically, there appears to be a suboptimal compensatory PEA response to neuropathic injury that leads to severe pain [31]. PEA appears to be able to reduce central sensitization and reduce pain severity [32]. In a rat oxaliplatin neuropathy model, PEA reduced pain hypersensitivity to mechanical and thermal stimuli. This was associated with the activation of PPAR-alpha. Histologically, microglia and astrocyte responses were dampened within the dorsal horn [33,34]. In Wistar rats, PEA prevented the oxaliplatin-induced activation of nuclear factor-kappa B and increased IL-1B and TNF-alpha expression. Histologically observed neuronal damage was reversed [34,35]. PEA reduces the allodynia experienced by paclitaxel-treated mice, potentially by activating PPAR-alpha [36].

PEA has been reported to be able to improve myelinated nerve fiber function in patients experiencing painful CIPN. In 20 patients undergoing thalidomide and bortezomib treatment for multiple myeloma, after a two-month treatment with PEA 300 mg BID using pain and warmth thresholds, blinded examiners measured motor and sensory nerve fiber function and laser-evoked potentials. They reported that pain and all neurophysiological measures significantly improved (*p* < 0.05) [37]. Another study involving patients receiving adjuvant or neoadjuvant paclitaxel or oxaliplatin for breast or colon cancers who had neuropathy after treatment reported that 70% had clinical stabilization or improvement in CIPN with PEA. Of note, this trial had no placebo arm [38].

PEA has been available as a dietary supplement in the United States since 2015. It is widely and rapidly distributed and has a short plasma half-life when taken by mouth. Due to hepatic recycling, two peaks occur at 70–90 min and 120–180 min [39,40,41]. PEA distribution is altered by tissue injury, with selective accumulation at injury sites [42,43]. PEA is metabolized by N-acylethanolamine acid amidase, fatty acid amide hydrolase, palmitic acid, and ethanolamine [44]. PEA has no known drug-drug interactions and has been reported to have no more significant side effects than a placebo [45]. Micronized and ultramicronized forms of PEA appear to have better oral bioavailability [45,46].

The primary objective of this present randomized trial was to look for evidence of the efficacy of PEA at two doses, relative to placebo responses, as a treatment for established CIPN. This trial was conducted to inform the design of a potential subsequent phase III PEA trial for treating bothersome established CIPN. The secondary objectives of this present trial were to assess the safety of PEA at the two study doses and to evaluate changes in patient-reported quality of life from baseline to the end of 8 weeks.

## 2. Methods

Eligible patients were adults with an ECOG performance status of 2 or better. Such patients need to have had pain, numbness, tingling, or other symptoms of CIPN for at least 3 months following completion of neurotoxic chemotherapy, with no further planned chemotherapy for at least 2 months after study registration. This study was limited to patients with taxane- and/or platinum-based neuropathy. Involved patients must have noted tingling, numbness, or pain symptoms of at least four out of ten within the week before study registration on a 0–10 scale where zero was ‘no problem’ and ten was ‘as bad a problem that could be imagined.’ Life expectancy must have been ≥6 months, and the patient could not have had evidence of residual cancer, per routine clinical practice-based parameters. They were not allowed to have had a previous diagnosis of diabetes or another non-chemotherapy-induced peripheral neuropathy. They could not have been using a cannabis product within the four weeks before registration. They could not have previously used PEA or be currently receiving or planning to start an opioid, duloxetine, gabapentin, or pregabalin.

After the patient was registered into the study, the values of chosen stratification factors were recorded regarding gender and the prior neurotoxic chemotherapy they had received (taxane-including regimens versus oxaliplatin-including regimens versus other). The patient was assigned to one of the two treatment groups (PEA versus placebo) and one of two dose levels (once or twice daily) using the Pocock and Simon dynamic allocation procedure, which balanced the marginal distributions of the stratification factors between the treatment groups. Beginning on day one of the study and for eight weeks, patients were instructed to take 400 mg of PEA or a matched placebo, either once or twice daily.

The PEA used in the present trial was OptiPEA^®^, obtained from Innexus Nutraceuticals in Nijmegen, The Netherlands. The PEA and placebo products were put into capsules that were indistinguishable from each other.

Treatment evaluation was conducted using three measures, all completed before treatment and at the end of every treatment week for eight weeks. The first of these was the EORTC QLQ-CIPN20 instrument [47,48,49,50]. Published literature provides evidence of its internal consistency and stability, reliability, sensitivity, convergent and contrasting group validity, and responsiveness; nine studies across multiple countries support its strong psychometric properties [51,52]. The second measure was the Patient Global Impression of Change (PGIC) tool, which came from the Clinical Global Impressions scale (CGI). The PGIC tool was taken from the CGI and adapted to be completed by patients, becoming a PRO measurement tool [53]. This tool was to be completed at the end of every treatment week. The third measure was the Chemotherapy-Induced Peripheral Neuropathy Assessment Tool, a validated measure [54,55] comprised of 14 items that might be affected by peripheral neuropathy. In this tool, patients were asked to complete a numerical rating of 0–10 related to neuropathy symptoms “not at all interfering” to “completely interfering”.

## 3. Statistical Considerations

The primary endpoint of this study was the change from baseline to eight weeks of the total score of the EORTC QLQ CIPN-20. The items of the CIPN-20 were linearly converted to a 0–100 scale so that a higher score corresponds to more symptoms. The change from baseline was calculated as the difference between the eight-week assessment and baseline. Patients who were eligible, randomized, initiated treatment, and completed the baseline questionnaire were considered evaluable for the analysis. For patients who did not complete all assessments, their scores were imputed using the last measure carried forward. Patients with no post-baseline data were considered to have no change from baseline. Differences in mean change from baseline to eight weeks between each active PEA arm and the combined placebo arms and the corresponding 95% confidence intervals were computed based on a two-sample *t*-test. The two placebo arms were combined and treated as one placebo arm for analysis purposes. Secondary and exploratory endpoints were scored according to their scoring algorithm and analyzed similarly to the primary endpoint analysis. Baseline characteristics were compared between treatment arms using a Kruskal–Wallis test or chi-square test [56,57]. The primary and secondary outcomes were compared between treatment arms using a Kruskal–Wallis test. Grade 3+ adverse events by patients were summarized by frequencies and severity using CTCAE v5.0.

Toxicity, quality of life, and cognitive function were primarily measured by patient-completed questionnaires addressing these topics. Patient baseline characteristics were compared between those who completed their eight-week assessment and those who did not complete their eight-week assessment. No differences were detected between the two groups, and the data were assumed to be missing completely at random. Three patients were excluded from the analysis for the following reasons: one patient had a significant treatment delay during their first week of treatment, one patient had a significant treatment delay during their second week of treatment, and one patient in the PEA 400 mg/d arm took twice their prescribed daily dose. A sensitivity analysis with these patients was conducted, and the results were maintained.

## 4. Results

**Figure 1:**
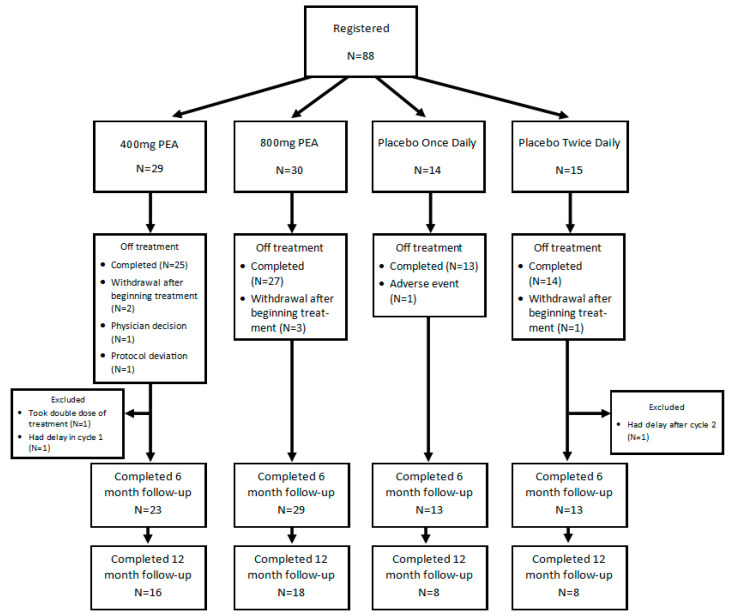
Consort Diagram.

A Consort Diagram (Figure 1) illustrates the study flow, which shows that more than 85% finished 8 weeks of treatment and that the number of patients completing the weekly CIPN-20 was 90% or greater. On-study characteristics are detailed in Table 1.

**Figure 2:**
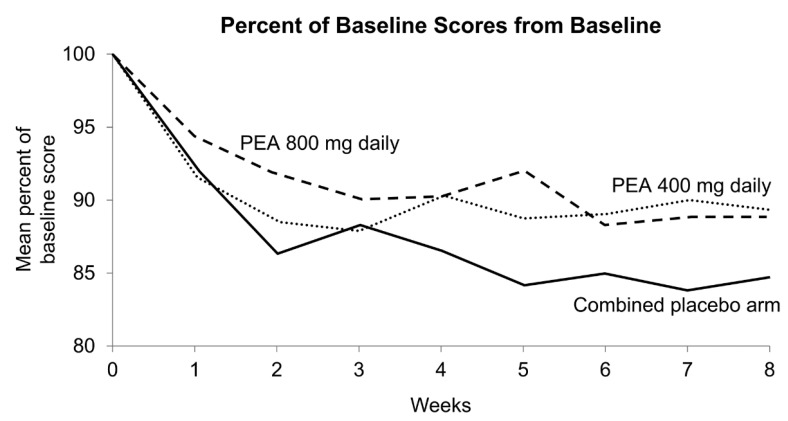
Percent of Baseline Scores from Baseline to Week 8 by Arm. Note that the placebo arm did numerically better than the two treatment arms, with a *p*-value of 0.11.

The primary result of this trial is illustrated in Figure 2, demonstrating that there was no suggestion that either of the PEA arms did any better than the placebo arm. There was no signal of significant toxicity differences between the three study arms. Quality of life outcome measures were similar between the study arms, as were cognitive function evaluations. More specifically, neither the Patient Global Impression of Change (PGIC) tool nor the Chemotherapy-Induced Peripheral Neuropathy Assessment Tool showed any suggestion of benefit for the PEA treatment arms. Patient-completed questionnaires at baseline and weekly during the trial regarding toxicity, quality of life, and cognitive function did not show any significant differences or trends between the three study arms.

## 5. Discussion

This study failed to find any suggestion of benefit for low- or higher-dose PEA in reducing established CIPN caused by taxanes or oxaliplatin. There was no suggestion of improvement in quality of life, nor was there any improvement in cognition. PEA was tolerated well. There was no signal of significant toxicity differences between the three study arms.

Chemotherapy-induced peripheral neuropathy is largely a sensory axonopathy and neuronopathy residing in the dorsal root ganglion, which is in response to an inflammatory reaction to chemotherapy agents [58,59]. PEA synthesis is stimulated in inflamed and damaged tissues, downmodulating neuroinflammation and reducing neuropathic pain. PEA has been shown to prevent nerve injury by inhibiting inflammatory infiltration in animal models [60]. This is mediated through the peroxisome proliferator-activated receptor (PPAR)-alpha [61]. Activation of PPAR-alpha suppresses the release of IL-1beta and IL-6 and blocks the up-regulation of cyclooxygenase. However, in pre-clinical studies, PEA was administered at the time of the chemotherapy and not after established nerve injury [34] or at the early onset of the neuropathy [36]. The current trial required patients to have established CIPN and to be at least three months following the completion of neurotoxic chemotherapy. The neurologic injury was likely established by three months, and any neuroinflammatory response would have been minimal. There, however, are animal data to support that PEA might treat existing CIPN and prevent CIPN. Given this, we decided to conduct this treatment trial first and subsequently consider doing a prevention trial [37].

When PEA is given concurrently with paclitaxel in vitro, it improves dorsal root ganglion neuron viability, protects neurite length, reduces neuron swelling, and protects neuron cell bodies [62]. Once the damage has occurred and the inflammatory response is resolved, the current data support that PEA does not appear to improve lost nerve function. The data from the current trial, supporting that PEA is ineffective in treating established CIPN, do not rule out the possibility that PEA may have benefits in preventing the development of CIPN when started with neurotoxic chemotherapy. This could be tested in a randomized, placebo-controlled trial.

Neuroinflammation from paclitaxel occurs not only in the spine but also in the hippocampus, which can lead to cognitive deficits and depression [63]. PEA has been shown to improve memory in healthy individuals in a randomized crossover trial comparing 600 mg of PEA with a placebo [64]. We did not find a subjective improvement in concentration or memory by asking patients to complete questions related to this topic, but no objective tests were conducted. It may be that PEA may prevent cognitive deficits associated with chemotherapy if started during chemotherapy rather than after cognitive deficits are established. A randomized trial would also be needed to answer this question.

## 6. Conclusions

In summary, PEA, though well tolerated, failed to improve the symptoms of established CIPN or subjective cognitive symptoms associated with prior chemotherapy. Future studies should explore using PEA at the start of neurotoxic chemotherapy. These studies could also explore subjective and objective cognitive outcomes.

## Figures and Tables

**Table 1:** Patient Demographics and Disease Characteristics.

	Arm				
	PEA 400 mg Daily (N = 29)	PEA 800 mg Daily (N = 30)	One Placebo Capsule Daily (N = 14)	One Placebo Capsule Twice a Day (N = 15)	Total (N = 88)
**Age**					
Median	67.0	60.0	56.0	68.0	63.5
**Ethnicity**					
Not Hispanic or Latino	27	27	12	15	81
Hispanic or Latino	1	1	2	0	4
Not Reported/Unknown	1	2	0	0	3
**Race**					
White	22	25	12	13	72
Black or African American	6	4	1	2	13
Not Reported/Unknown	1	1	1	0	3
**Gender**					
Male	5	6	3	3	17
Female	24	24	11	12	71
**Prior Chemotherapy**					
Any taxane +/− carboplatin +/− other agents	21	21	10	11	63
Oxaliplatin based	8	8	4	4	24
Cisplatin without another neurotoxic agent	0	1	0	0	1
**BMI**					
Mean (SD)	32.1	31.3	35.1	31.0	32.1
**BMI Category**					
Normal	6	6	1	4	17
Overweight	8	8	3	3	22
Obese	15	16	10	8	49
**ECOG PS**					
0	14	14	8	7	43
1+	15	16	6	8	45

## Data Availability

The original contributions presented in this study are included in the article. Further inquiries can be directed to the corresponding author.
